# Integrating the metabolic and molecular circuits in diabetes, obesity and cancer: a comprehensive review

**DOI:** 10.1007/s12672-024-01662-1

**Published:** 2024-12-18

**Authors:** Shrikirti Anand, Trupti N. Patel

**Affiliations:** Department of Integrative Biology, School of Bio-Sciences and Technology, Vellore Institute of Technology, Vellore, Tamil Nadu 632014 India

**Keywords:** Diabetes, Obesity, Diabesity, Cancer, Signaling pathways, DNA damage and repair

## Abstract

The progressive globalization of sedentary lifestyles and diets rich in lipids and processed foods has caused two major public health hazards—diabetes and obesity. The strong interlink between obesity and type 2 diabetes mellitus and their combined burden encompass them into a single term ‘Diabesity’. They have also been tagged as the drivers for the onset of cancer. The clinical association between diabetes, obesity, and several types of human cancer demands an assessment of vital junctions correlating the three. This review focuses on revisiting the molecular axis linking diabetes and obesity to cancer through pathways that get imbalanced owing to metabolic upheaval. We also attempt to describe the functional disruptions of DNA repair mechanisms due to overwhelming oxidative DNA damage caused by diabesity. Genomic instability, a known cancer hallmark results when DNA repair does not work optimally, and as will be inferred from this review the obtruded metabolic homeostasis in diabetes and obesity creates a favorable microenvironment supporting metabolic reprogramming and enabling malignancies. Altered molecular and hormonal landscapes in these two morbidities provide a novel connection between metabolomics and oncogenesis. Understanding various aspects of the tumorigenic process in diabesity-induced cancers might help in the discovery of new biomarkers and prompt targeted therapeutic interventions.

**Graphical Abstract:**

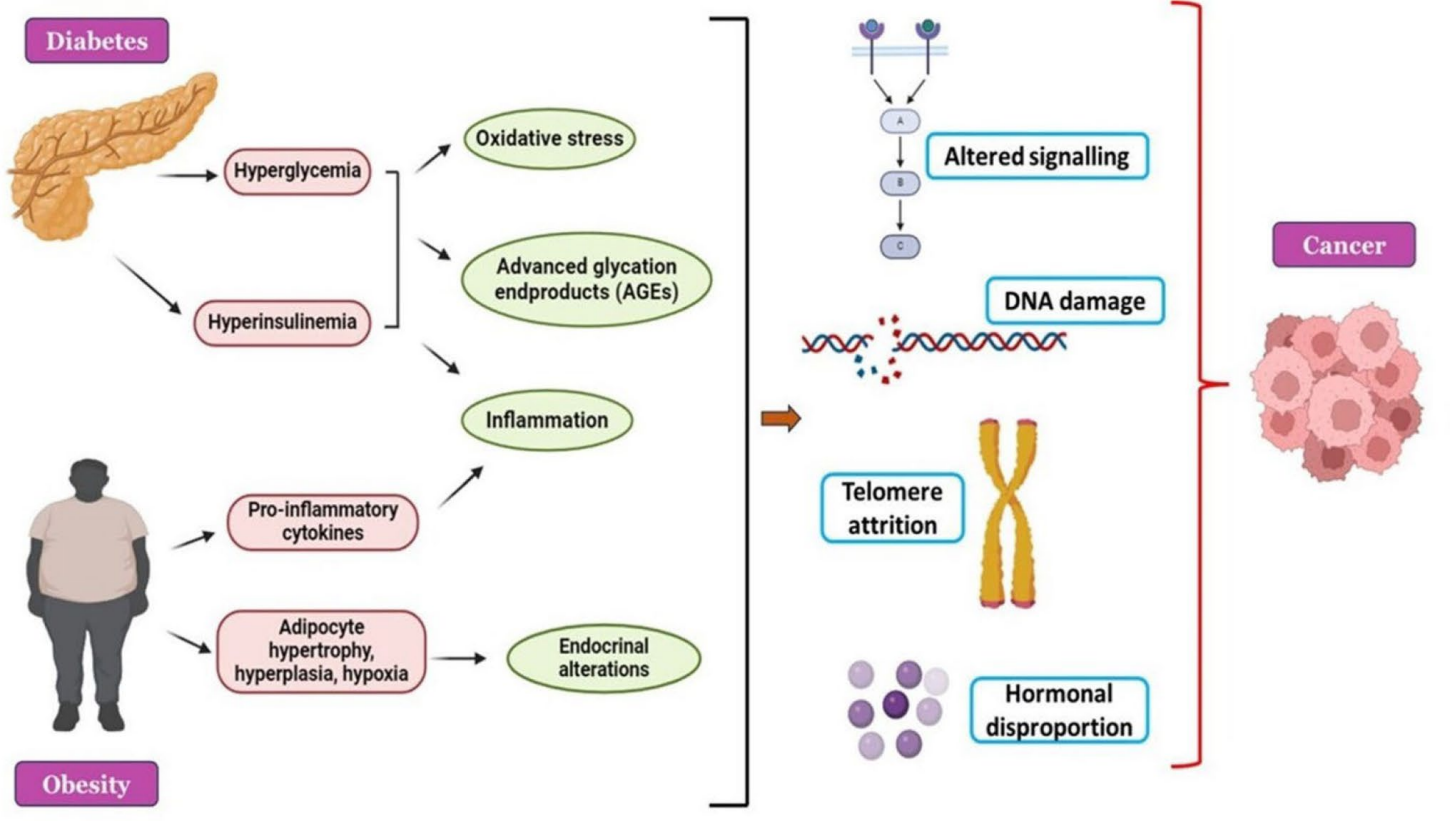

## Introduction

The rampantly occurring conditions of Diabetes and Obesity over the last years represent an impressive risk to public health labeling them as the ‘twin epidemics’ [1]**.** The global prevalence of diabetes is estimated to be 422 million people while the obesity web has enlisted 890 million adults as well as 160 million adolescents, and children worldwide [2, 3]. It is worth noting that over 80% of diabetes patients are also obese [4]. Excess body weight and obesity are significant risk factors for type 2 diabetes mellitus (T2DM) [5]. These metabolic syndromes together known as ‘Diabesity’ work as two sides of the same coin and have morbid consequences like cardiovascular conditions (CVDs), the leading cause of death worldwide, and cancer, the grossly incurable fearful condition gripping 39% of the world population [6, 7]. Independently, diabetes and obesity have been shown to increase the risk for various tumors with the strongest correlations identified for endometrial, colorectal, pancreatic, hepatic, and post-menopausal breast cancer [8]. Despite the healthcare and socio-economic burden posed by diabesity, the mechanisms whereby diabetes and obesity favor cancer development remain largely under-investigated.

The pathophysiology of diabetes mellitus (DM) is attributed to a state of continued high blood glucose levels arising from inadequate amounts of insulin produced by the body or due to the inability of insulin to act on cells, a process also known as insulin resistance. Obesity is characterized by the accumulation of excessive amounts of body fat inducing a constellation of metabolic catastrophes including chronic low-grade inflammation, insulin resistance, and atherogenic dyslipidemia (↓HDL-cholesterol with ↑ triglycerides in plasma) [9, 10]. Many molecular disruptions in diabesity suggest that various essential cellular and biochemical cascades possibly change their conventional course under metabolic upheaval. These diverted pathways are crucial drivers of cancer cell proliferation and metastasis. Moreover, poorly regulated concentrations of insulin, IGF, inflammatory cytokines, and hormones like leptin and estrogen in individuals with diabetes and obesity disrupt the DNA helix and refashion the DNA damage response mechanism, causing genomic instability, a hallmark of cancer [11, 12]. Another molecular culprit that has been associated with diabetes, obesity, and cancer is telomere. Various pieces of evidence have pointed out that specific lifestyle factors such as obesity can increase the pace of telomere shortening which contributes to the early onset of age-related disorders such as CVDs, diabetes, and cancer [13–15].

Pathologies of the three diseases are intertwined in a complex manner with various metabolic players of diabesity determining critical interactions associated with the onset of neoplasms. The purpose of this review is to shed light on various metabolic and molecular axes that link “the hazardous trio” and unravel the irregularities that occur in people with diabetes and obesity making them susceptible to tumorigenesis.

## Pathways intervening in diabetes, obesity, and cancer

### Insulin signaling pathway

**Fig. 1:**
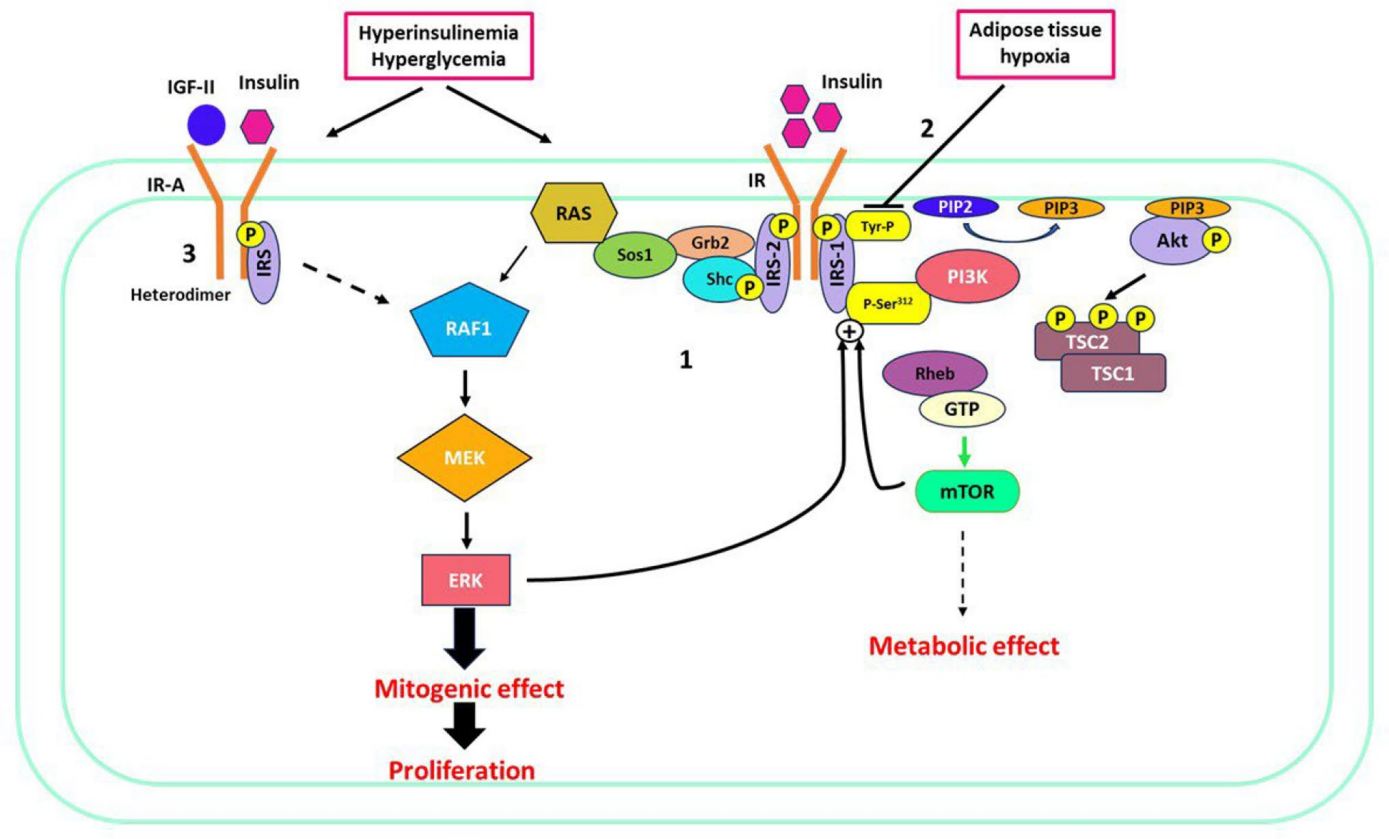
The anomaly of the insulin-signaling pathway. 1-Hyperinsulinemia favors the mitogenic branch and attenuates the metabolic branch of the insulin cascade driving proliferation for cancer cells. 2-Adipose tissue hypoxia inhibiting IRS by preventing autophosphorylation of tyrosine residue in IRS1. 3-Hyperglycemia facilitates the IR-A receptor expression favoring the formation of insulin-IGF-II heterodimer, further activating the MEK/ERK signaling

The paradoxical role of the insulin signaling pathway in insulin-resistant and hyper-insulinemic subjects brings it to the crossroads of diabetes, obesity, and cancer. Insulin binds to the insulin receptor (IR) to modulate a wide range of downstream signaling pathways that affect apoptosis, cell growth and proliferation, differentiation, and metabolic circuits [16, 17]. This pathway diversifies into two branches – the metabolic pathway (PI3K/AKT/mTOR) and the mitotic pathway (RAS/RAF/MAPK/ERK). In normal-insulinemic individuals, target tissues respond to insulin by activating the PI3 kinase pathway exerting metabolic effects. On the contrary, IR signaling for the metabolic branch is blunted in hyper-insulinemic subjects while the mitogenic branch remains unaffected [18]. Abnormal phosphorylation of serine 312 in insulin receptor substrate 1 (IRS1) in insulin-resistant subjects inhibits recruitment and activation of PI3K which generates a negative feedback loop for further attenuating metabolic activity. Inactive PI3K renders Akt inactive leading to the overactivation of mTORC1. Unlike metabolic attenuation, hyperinsulinemia increases ERK activation instead of decreasing it. Overactivation of the mitogenic branch is also attributed to increased IRS2 expression. Elevated IRS-2 expression results in either unchanged or greater recruitment of Grb2, increased expression of Raf-1, and ultimately higher ERK activation. This in turn, enhances phosphorylation of Serine-312 on IRS-1, thereby inhibiting it (Fig. 1) [19]. Adipose tissue hypoxia in obesity also reduces IR and IRS1 tyrosine autophosphorylation, directly affecting their ability to perceive insulin [20]. Inhibitory effects on IRS1 activity further favor the mitogenic branch of the insulin signaling pathway. The distinct roles of IRS1 and IRS2 proteins have also been studied in human skeletal muscles whereby IRS1 is principally engaged in glucose metabolism while IRS-2 is largely associated with the activation of MAPK/ERK pathway [21]. Overstimulation of MAPK/ERK signaling also results in prolonged activation of FTase, leading to an elevated level of farnesylated Ras. These elements further enhance the mitogenicity of other growth factors, facilitating cancer advancement [22]. The selective up-regulation of the insulin mitogenic pathway as a result of hyperinsulinemia offers compelling evidence for the promotion of cancer growth in individuals with diabetes. The two isoforms of insulin receptor, A and B are also responsible for maintaining the balance between metabolic and mitogenic pathways. IR-A is preferentially expressed in cancer cells while IR-B is mostly expressed in tissues such as adipose tissue, liver, and muscle, and regulates metabolic homeostasis [23]. IR-A because of its ability to heterodimerize with IGF-II, systemic levels of which are elevated in obese and diabetic individuals, promotes cell proliferation and invasion in tumor progression (Fig. 1). Chronic hyperglycemia has shown a correlation with an increased IRA/IRB ratio in human pancreatic islets. Weight loss in people with obesity results in an increase in IR-B in adipose tissue and adipocytes [24]. The pathophysiological processes in diabesity facilitate mechanisms such as preferential overexpression of IR-A and impairment in IRS 1 function which are exploited by the cancer cells to drive their proliferation [25].

### Adrenergic signaling

**Fig. 2:**
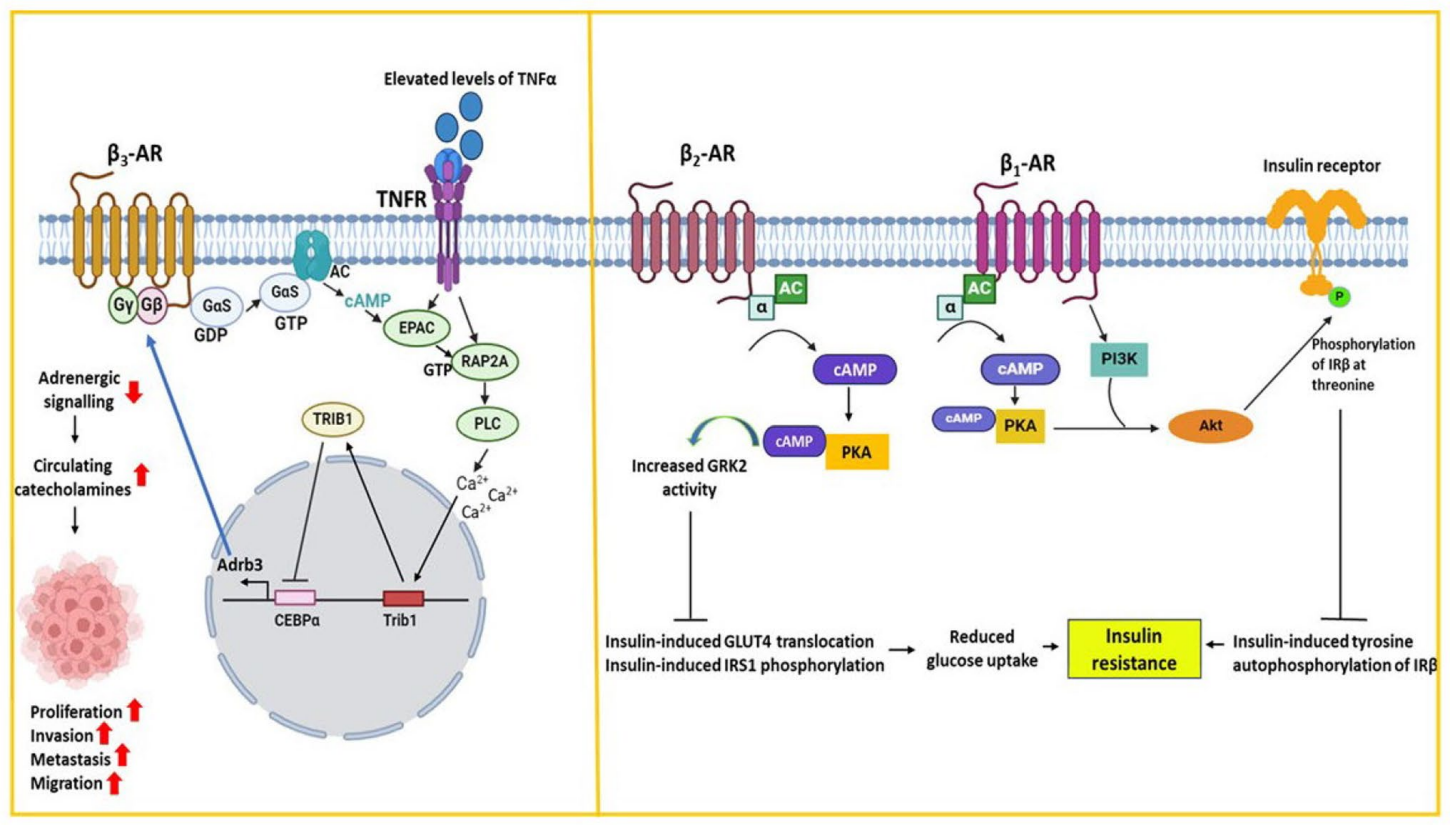
Adrenergic signaling at the crossroads of diabetes, obesity, and cancer. Inflammatory pathways in obesity activate the EPAC/RAP2A/PLC pathway to depress β3-adrenergic signaling leading to catecholamine resistance. Elevated levels of catecholamines trigger malignant features (Right panel). Overstimulation of β1-AR and β2-AR inhibits the insulin signaling pathway and leads to insulin resistance contributing to diabetic phenotype (Left panel)

Adrenergic signaling regulates the fight-or-flight response in our body by stimulating G-protein-coupled receptors with their ligands, catecholamines—epinephrine and norepinephrine. Diverse adrenergic receptors (AR) exhibit tissue-specific expression and communicate via distinct metabolic pathways [26, 27]. These molecules can activate several pathways depending on the G-protein they are coupled with, with the most significant one being the cAMP signaling cascade, the activation of which induces the enzymatic reaction of adenylate cyclase (AC) and the formation of cAMP. Activation of cAMP stimulates effector proteins leading to the transcriptional regulation of several genes especially cascades associated with proliferation, metabolism, DNA damage and response, inflammation, and other processes that are pertinent to diabetes, obesity, and cancer [28, 29]. β-adrenergic signaling plays a significant role in adipose tissues whereby it regulates body weight [30]. The expression of beta-adrenergic receptors is significantly decreased in adipose tissues of many mice models exhibiting obesity [31]. It is well established that increased inflammation and elevated cytokine levels are characteristic traits of both obesity and T2DM. These contribute to insulin resistance and are also associated with catecholamine resistance [32]. Elevated levels of inflammatory cytokine TNF-α downregulate β-adrenergic receptor response to catecholamines, thus reducing cAMP levels responsible for increasing energy expenditure in adipose tissues [33]. Among the three β-ARs, β3- adrenergic receptors are by far the most expressed form in adipocytes and its activation is the rate-limiting step in adipocyte catecholamine signaling. Downregulation of β3- adrenergic signaling is likely the key molecular event in catecholamine resistance wherein adipocytes in obese individuals cannot respond to catecholamines to the same extent as non-obese ones. Elevated levels of TNF-α are documented to cause heterologous desensitization of Adrb3, the gene encoding the β3- adrenergic receptor which is responsible for catecholamine resistance in adipocytes. This negative regulation of Adrb3 transcription by TNF-α is achieved by utilizing the EPAC/RAP2A signaling. TNF-α induces EPAC and RAP2A, both of which trigger PI-PLC to elevate cytosolic calcium ions that in turn cause the stimulation of pseudokinase TRIB1. TRIB1 further promotes the proteasomal degradation of CEBPα, the primary transcriptional activator of Adrb3 [34]. Catecholamine resistance leads to increased levels of circulating catecholamines and has a growth-promoting effect on tumor cells (Fig. 2). Elevated levels of epinephrine and norepinephrine have been associated with severe gastric carcinoma and poorer survival [35]. Catecholamines also facilitated the advancement of ovarian cancer through the secretion of CXC chemokines [36]. Norepinephrine treatment is reported to increase invasive capacity in breast cancer cell lines [37]. Hence, diabesity-induced inflammation depresses β3-adrenergic signaling contributing to catecholamine resistance which is likely to increase the risk of incidence of tumors. Sustained and overstimulation of β1- and β2- adrenergic receptors are key regulators of glucose homeostasis and have been widely associated with insulin resistance in cardiac muscles. Stimulation of β2-AR by non-selective agonist isoproterenol leads to AC activation and cAMP generation that activates PKA which in turn phosphorylates and increases GRK activity suppressing insulin-mediated GLUT4 expression and translocation. In β1-AR signaling, PKA and PI3K activate Akt by phosphorylating it at Ser 473, furthur phosphorylating the β subunit of insulin receptor (IRβ). This prevents insulin-induced tyrosine autophosphorylation of the IRβ receptor, resulting in a disruption of insulin signaling. This cross-talk between insulin and β2- adrenergic signaling system is crucial in the pathogenesis of insulin resistance [38]. The involvement of adrenergic signaling in different molecular events of diabetes and obesity contributing to cancer highlights its role in linking the three disorders.

### Wnt/β-catenin signaling pathway

**Fig. 3:**
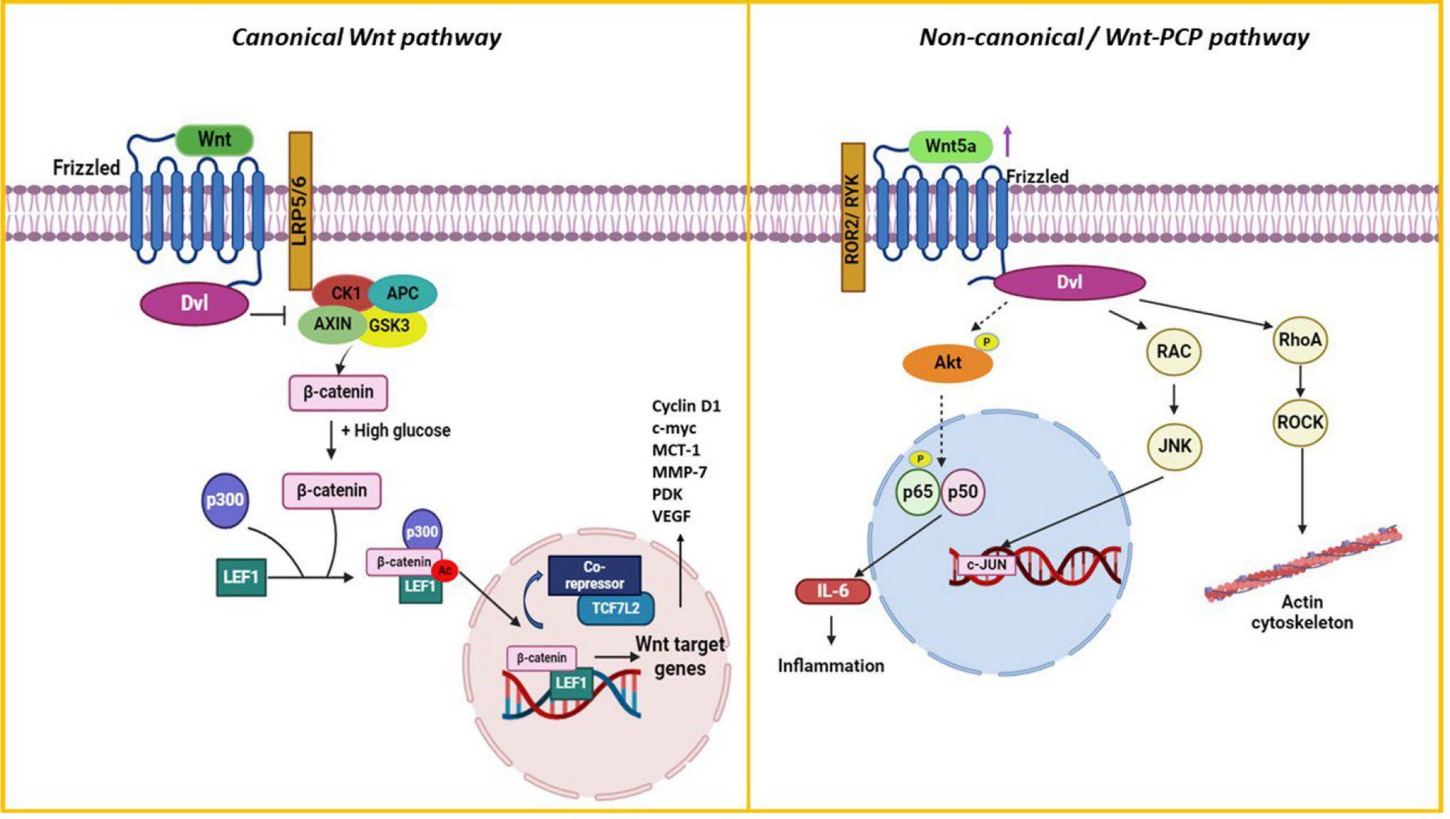
High glucose in diabetes enhances Wnt-stimulated nuclear accumulation of β-catenin. The concurrent presence of both WNT and high glucose is required to express WNT target genes (Left panel), contributing to tumorigenesis. Increased expression of Wnt5a in obesity increases IL-6 levels, activates the JNK pathway, which is involved in the progression of insulin resistance, and regulates cytoskeleton remodeling exploited by the cancer cells to sustain their proliferation (Right panel)

The classical Wnt/β-catenin signaling is implicated in developing diabetes, obesity, and cancer by controlling inflammation and insulin resistance, making it an intersecting node between metabolic disorders and cancer. Abnormal Wnt signaling is implicated in numerous cancer entities including the pathogenesis of pancreatic cancer whereby it has been reported to maintain cancer stem cells and promote resistance to apoptosis [39]. The two branches of Wnt signaling – Canonical (β-catenin-dependent) and Non-canonical (β-catenin-independent) pathways not only activate distinct intracellular signaling cascades but also have varied roles in maintaining an inflammatory/anti-inflammatory profile in obesity [40]. Activation of canonical Wnt signals inhibits the accumulation of inflammatory cytokines like TNF-α and IL-6. However, non-canonical Wnt ligands such as Wnt5a expression have been correlated with markers of inflammation in visceral adipose tissue. They are reported to increase IL-6 production, thus associating it with systemic inflammation and visceral adiposity [41]. Wnt5a is highly expressed in adipocytes when the diet contains fatty foods. Overexpressed Wnt5a along with Wnt5b and Wnt4, facilitates the stimulation of adipogenesis; however, excessive levels of these factors hinder adipocyte differentiation, increasing hypertrophic fat cells. Additionally, enlarged adipocytes in the obese state have shown increased secretion of Wnt5a that could impede insulin signaling and develop glucose intolerance by activating the non-canonical Wnt5a/PCP pathway. In the WNT/PCP pathway, Wnt5a forms a complex with the Frizzled receptor (FZD), ROR2, and Ryk, which stimulates the Dishevelled (Dvl), subsequently activating DAAM1, RhoA, and eventually ROCK and MRLC. Dvl can also activate the Rac1 protein, which causes the Filamin A protein and JNK to phosphorylate c-JUN and translocate it to the nucleus. This downstream protein activation has been linked to cell migration, invasion, and F-actin cytoskeleton remodeling [42] (Fig. 3). This highlights the association of Wnt5a with cancer progression and development [43]. Wnt-5a knockdown in PANC-1 pancreatic cells modulated the cell cycle while its expression in MCF-7 breast cells was related to migration [44]. The canonical Wnt signaling, on the other hand, establishes a connection between hyperglycemia and cancer. In the β-catenin dependent signaling, WNT molecules attach to frizzled and lipoprotein receptor-related protein LRP5/6 coreceptors. This inhibits the “destruction complex” consisting of APC, CK1, AXIN, and GSK3 protein, which in the absence of Wnt phosphorylates cytosolic β-catenin to trigger its degradation [40]. Formerly, it was believed that stabilized β-catenin is translocated from cytosol to nucleus. It would then operate as a potent co-activator for transcription factor members of the TCF/LEF family, such as TCF7L2 and LEF1, activating target genes involved in pro-survival and proliferation. Recent research provides conclusive evidence that WNT activation alone does not promote the nuclear accumulation of β-catenin. However, this mechanism depends on and requires high glucose levels to boost its nuclear retention. High glucose levels and the activation of wnt proteins work together to induce acetylation of lysine residues in the armadillo repeats of β-catenin by p300. The levels of p300 are elevated by both wnt and high glucose. The process of acetylation is necessary for the creation of the LEF1-β-catenin complex. This complex enables the entry of β-catenin into the nucleus and allows for selective DNA targeting by the LEF1-β-catenin complex on the Wnt target genes after displacing the TCF7L2-corepressor complexes [45]. Among the Wnt target genes include cyclin D1, c-myc, cyclin E, PDK, VEGF, fibronectin and MCT-1, and MMP-7, which are involved in various hallmarks of cancer such as cell cycle progression, angiogenesis, and epithelial-mesenchymal transition (EMT) [46] (Fig. 3). Expedited levels of LEF-1 in tumors coupled with high glucose levels in diabetic individuals promoting the LEF1–β-catenin complex formation is highly likely to accelerate malignant transformations, hinting at the prominent role of Wnt signaling in diabetes and cancer. Abnormal expression of the transcription factor LEF1 has been associated with many types of tumors including colonic adenocarcinoma. Downregulation of LEF1 inhibited the malignancy and motility-related microstructures including the polymerization of β-tubulin, F-actin, and Lamin B1. Suppression of LEF1 also resulted in the downregulation of genes associated with EMT indicating that LEF1 has a role in inducing carcinogenesis [47]. Rechanneling of the Wnt/PCP pathway and high glucose amplification of the classical Wnt pathway in obese and diabetic environments modify the cancer-related Wnt/ β-catenin signaling.

## DNA damage as an ontological link between diabetes, obesity, and cancer

**Fig. 4:**
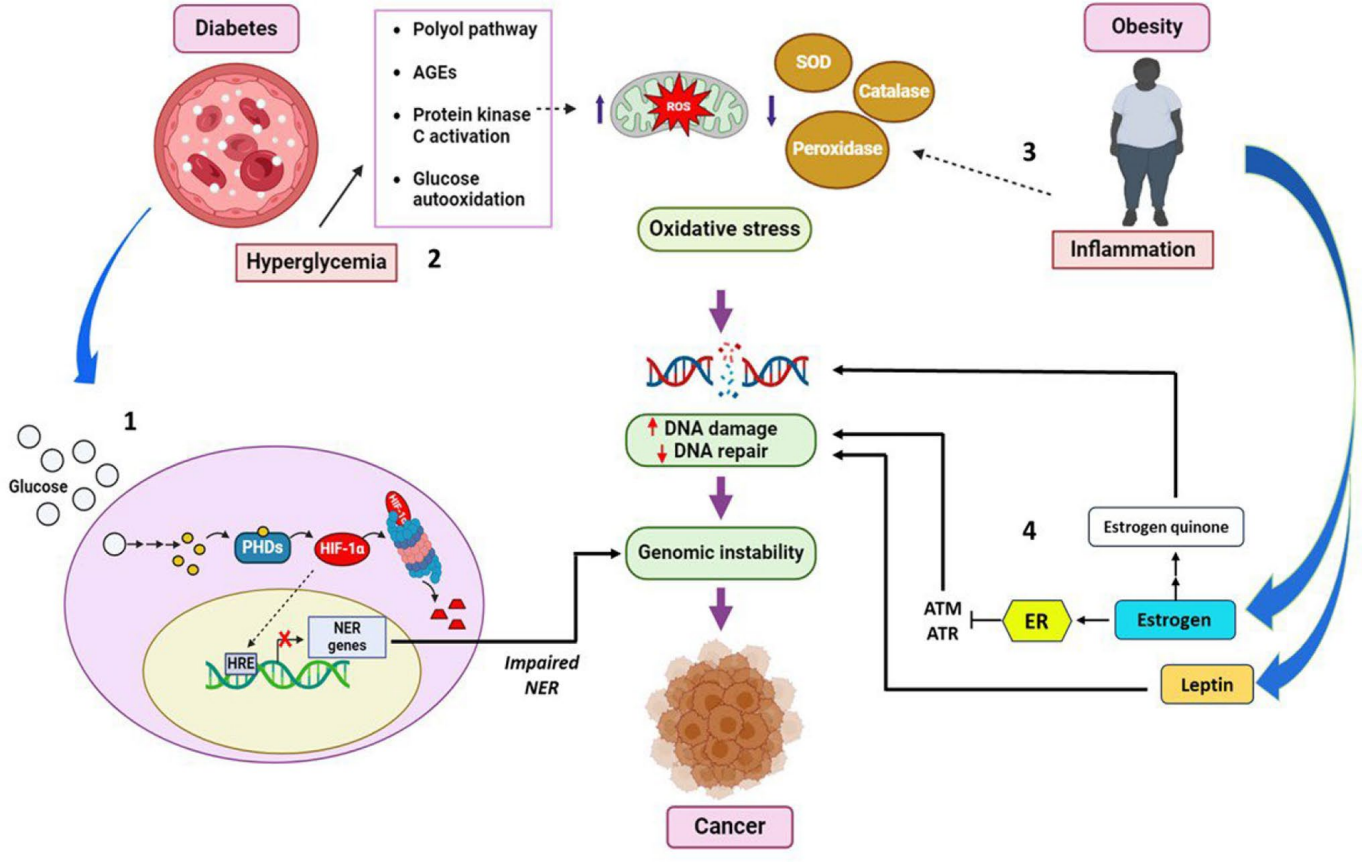
DNA damage as the ontological link between diabetes, obesity, and cancer. 1-High glucose inhibits the nucleotide excision repair (NER) pathway leading to a compromised DNA repair mechanism 2- Hyperglycemia triggers the overproduction of ROS leading to oxidative stress which causes DNA damage 3-Chronic inflammation in obese individuals disrupts the redox balance contributing to oxidative stress-induced DNA damage 4-Elevated levels of leptin and estrogen in obesity induces DNA damage. Estrogen binds with its receptor estrogen receptor α (ER) to inhibit the DNA damage responders—Ataxia telangiectasia mutated (ATM) and ATM- and Rad3-related (ATR) kinases

Accumulation of DNA damage and dysregulation of DNA damage response(DDR) has been implicated in the onset of metabolic diseases like diabetes and obesity as well as correlates with pathophysiological states of cancer. DNA lesions can arise due to metabolic upheaval in diabesity challenging the genomic integrity. Accelerated inflammatory response and associated oxidative stress drive DNA damage in people with diabesity [48]. A positive energy balance i.e. energy intake surpassing the energy expense in individuals with obesity causes clinical hypertrophy and hyperplasia of adipocytes and reduces oxygen availability making them hypoxic. The enlarged adipocytes become the source of pro-inflammatory cytokines including CRP, TNF-α, and interleukins (IL-1, 6, 8, 10, 15, and 18). These adipocytokines recruit immune cells like macrophages, neutrophils, and dendritic cells to adipose tissue, predominantly the white adipose tissue (WAT) [49]. Being the primary responders of caloric excess, WAT is then infiltrated by M1 proinflammatory macrophages which are the chief sources of TNF-α and IL-6 [50]. These are believed to cause low-grade chronic inflammation – the pathological state underlying obesity. These pro-inflammatory cytokines induce the production of ROS and nitrogen [51, 52]. Hyperglycemic states in diabetics also participate in mitochondrial ROS production via four major mechanisms viz., activation of PKC, hexosamine and polyol pathways, glucose autooxidation, and protein glycation resulting in the generation of advanced glycation end products **(**Fig. 4**)** [53]. Increased ROS and free radicals impair the cellular redox homeostasis triggering oxidative stress. This is characterized by an imbalance between diminished amounts of antioxidant scavengers like glutathione peroxidase, catalase, and superoxide dismutase, and elevated concentrations of oxidants such as hydrogen peroxide (H_2_O_2_), superoxides (O_2_^−^), and hydroxide (OH^−^) radicals.

Endogenous bombardment of ROS and free radicals damages the DNA causing single and double-strand breaks, abasic sites, and base modifications. Maintenance of genomic homeostasis requires the removal of damage accumulation and error-free repair mechanisms. In a pioneering study by Douglas and Hanahan, genome instability has been defined as a prominent hallmark of cancer wherein the remarkable capacity of genome maintenance systems to detect and correct errors in DNA is impaired generating random mutations and allowing cancer cells to proliferate and disseminate [54, 55]. The accumulation of DNA lesions and dysregulated DDR in diabesity cause genetic instability predisposing normal cells to malignant transformations. Free radicals in individuals with diabetes are known to induce double-strand breaks (DSBs) in the sugar-phosphate backbone increasing chromosomal anomalies such as an increase in frequency of sister chromatid exchange (SCE) and micronucleus (MN). In a study conducted on T2DM-afflicted individuals, the SCE and MN frequency was significantly higher than the control group [56]. Another study revealed that the number of DNA strand breaks in diabetes patients is positively associated with fasting blood glucose and HbA1c (Glycated hemoglobin) and that the amount of strand breaks is substantially higher in diabetics than in controls [57]. Szymczak and colleagues conducted a comparative study between individuals with nondiabetic and diabetic colorectal cancers (CRCs), finding that patients with both T2DM and CRC exhibited the highest levels of endogenous oxidative DNA damage in their lymphocytes. The efficacy of DNA repair was reduced in CRC patients with T2DM, whereas it returned to normal levels in control patients within two hours [58]. Increased levels of cancer biomarkers such as CA-125, AFP, PRL, and CEA were found in T2DM subjects as compared to non-diabetic control in a case-controlled study. Higher levels of 8OHdG, the most abundant endogenous mutagen and a marker of oxidative stress, showed a positive correlation with HbA1C in T2DM individuals [59]. High-glucose environments also induce genomic and mitochondrial DNA damage in kidney podocytes disrupting the slit membrane and causing proteinuria [60].

Clear effects of DSBs were seen in the livers and colons of obese rats as evaluated by increased ϒ-H2AX detection [61]. Obese and lean rats treated with DMBA showed different levels of oxidative damage (8-OH-guanosine) and a modification of the GSH/GSSG ratio (a sign of oxidative stress) in the liver, of which mice with obesity showed more liver damage than the lean one [62]. A comet assay demonstrated that excessive body fat leads to elevated single and double-strand breaks and apurinic sites supporting the notion that obesity causes excessive DNA damage [63]. Visceral fat accumulation in obesity also induces lipid peroxidation and damage throughout the body due to excess free fatty acids which further triggers systemic oxidative damage. Recent reviews detail the production of DNA adducts by lipid peroxidation (LP) products such as 4-HNE, MDA, 8-oxo-Dg, and acrolein [64, 65]. Elevated levels of various LP indicators have been repeatedly seen in the blood, muscles, and adipose tissue of obese persons [66]. Serum levels of MDA and 4-hydroxyalkenals and the presence of 8-oxo-dG in blood have been related to BMI and hip circumference, establishing a connection between obesity-induced LP indicators and DNA instability in obese subjects.

Modulations in DNA damage response primarily due to changes in the expression of genes involved in DNA repair have also been observed in the disrupted metabolic homeostasis states of diabetes and obesity. Gene expression investigations using microarrays have revealed downregulation of DNA repair genes in T2DM subjects compared to healthy controls [67]. Hyperglycemia is also known to modify the NER mechanism in T2DM individuals. Chronic high glucose causes elevated amounts of 2-ketoglutarate and PHD thus repressing HIF-1α. Many NER genes like XPA, XPC, and CSA, CSB have hypoxia response elements in their promoters and require HIF-1α for activation. Repression of HIF-1α therefore impairs the NER mechanism causing replication stress and DNA strand breaks **(**Fig. 4**)** [68]. Diminished DNA repair activity due to obesity has been proved by remarkable tests on mice, in which the offspring fed on a high-fat diet detected a decrease in the BER gene, OGG1 in hippocampus areas while the mothers on a folate diet showed no significant change [69]. Loss of achieving net balance in establishing cellular damage and response homeostasis in diabetes and obesity pathologies appears to result in genomic instability and can be indicative of long-term carcinogenic consequences.

## Hormonal effervescence in diabesity and cancer

Altered hormonal landscapes within people with diabetes and obesity have painted a complex picture of the adverse impacts of these hormones in neoplastic transformations. The unregulated endocrine function of adipose tissue in individuals with obesity leads to the dysregulated release of various adipokines, including leptin, ghrelin, and resistin. Leptin is vastly responsible for DNA damage-induced cancers. Elevated levels of pro-inflammatory leptin have been found to cause DNA damage in breast epithelial cells [12]. Treatment of BRCA1 and BRCA2 heterozygous breast cancer cell lines with leptin for 24 h induced significant DNA damage [70]. Excessive leptin can stimulate the production of ROS, mostly through the activation of NADPH oxidase [71]. Moreover, binding of leptin to one of the isoforms of the leptin receptor induces the activation of multiple signaling pathways including the PI3K, JAK/STAT, and MAPK pathways, which ultimately promote cell proliferation. Resistin has a dual effect: it causes insulin resistance by blocking glucose transport leading to diabetes, and it is also believed to stimulate the proliferation of breast cancer cells by activating TLR4-mediated activation of NF-κβ and STAT3 [72]. Primarily recognized as an appetite stimulant, ghrelin has lately been identified as a putative regulator in the diabetes-obesity-cancer axis. It regulates UCP2 in pancreatic β cells and inhibits the release of insulin contributing to the development of T2DM. The ln-1 ghrelin splice variant is highly expressed in pituitary, prostate, breast, and neuroendocrine tumors and has been associated with tumor aggressiveness. A positive correlation exists between In1-ghrelin and Ki67 expression, a key cell proliferation marker. Increased expression of the GOAT enzyme, a member of the ghrelin family, was found in prostate cancer patients with dyslipidemia, overweight/obesity status, and/or diabetes [73]. Adipose tissue is the main source of estrogen in postmenopausal women. Enlarged adipocytes in overweight postmenopausal females facilitate high circulating levels of estrogen which fuels the pro-proliferative impact of these steroids causing accumulation of replicative errors leading to mutations and breast cancer progression [74]. Further, the heightened accessibility of estrogen amplifies the process of converting estrogen into estrogen quinone which forms bulky adducts with nucleic acids. Estrogen receptor-α upon binding to its substrate further decreases the activity of ATM and ATR, transducers of DNA damage response (Fig. 4). Their downregulation further hinders the effectors and suppresses mediators like Ku70/80 disturbing NHEJ [75]. Alterations in thyroid hormone (TH) levels have also been linked to the association between DM and cancer. THs reduce circulating triglycerides and cholesterol‐containing lipoproteins and are also responsible for stimulating GLUT4 expression, the plasma membrane glucose transporter mediating glucose uptake in skeletal muscles, thus representing a direct link with carbohydrate metabolism. Hyperthyroidism is linked to insulin resistance and dyslipidemia and clinical evidence indicates that it is also associated with higher risks of developing thyroid, prostate, and breast cancer [76]. The association of hormonal imbalance with different aspects of the pathogenesis of metabolic disorders and cancers suggests that they contribute to certain shared mechanisms between diabetes, obesity, and cancer.

## Telomere, diabetes, obesity and cancer

Over the last decade, there has been incredible growth in understanding different aspects of telomere biology in human disease. Telomeres are conserved tandem repeats at chromosome ends with more than 2000 repeats of ‘TTAGGG’ sequences that play critical functions in maintaining chromosomal integrity [77]. In normal somatic cells, a portion of this far-end chromosomal sequence is lost after each cell division, and upon reaching a critical length, telomeres are identified by DNA repair machinery. This inhibits subsequent cellular division resulting in either apoptosis of the cell or a state of irreversible cell cycle arrest known as replicative/cellular senescence [78, 79]. Telomeres are lengthened by the activity of telomerase enzyme which consists of two components—an RNA component acting as a template to the telomeric sequence and a catalytic subunit, a reverse transcriptase (TERT) synthesizing new telomeres having its RNA as a template. The cell in this manner compensates for telomeric loss continuing its divisions. Nevertheless, immortality not being a human trait, telomerase is not active in mature cells ultimately resulting in the death of cells [80, 81]. In contrast, tumor cells have an adaptive mechanism to maintain a critically short telomere length over successive cell divisions and acquire replicative immortality – a hallmark of cancer [82]. This is achieved by the reactivation of telomerase, a major route by which tumor cells prevent complete telomere erosion enabling the continuous proliferation of genetically unstable cancer cells. In such cases, telomeres become dysfunctional forming anaphase and chromatin bridges [83, 84]. Telomerase reactivation is integral to the telomere maintenance mechanism, enabling cancer cells to induce genetic instability while maintaining cellular viability [85]. The influence of diabetes and obesity on this hallmark of cancer is contentious. Nonetheless, these metabolic disorders represent conditions characterized by persistent low-grade inflammation, thereby linking them to the regulation of telomerase activity [86]. As one illustration, various proteins have converging molecular pathways in both inflammation and telomerase expression. NF-κB, the prime inflammatory pathway, has been demonstrated to regulate telomerase expression whereas RAP1, a shelterin protein involved in telomeric lengthening is implicated in NF-κB signaling pathways [87]. Elevated adipokines in obesity promote telomerase activity in specific cases. Leptin has been found to upregulate telomerase activity as well as mRNA and protein levels of TERT in human breast cancer cells [88, 89]. Resistin increases the expression of hTERT in gastric cancer cells [90, 91]. At present, the link between replicative immortality and the diabesity state is sparse.

Several other studies report the presence of shorter telomeres in individuals with diabetes and obesity predisposing cells to genomic instability [92, 93]. Shortened telomeres were first observed in the white blood cells of T2DM patients [94]. A shorter relative leukocyte telomere length was substantially related to an elevated risk of hyperglycemia progression in individuals diagnosed with T2DM [95]. Obese women have been reported to have significantly shorter telomeres compared to lean women [13]. An inverse association has been observed between telomere length and adiposity indices such as BMI, waist-to-height ratio, total body fat, and waist circumference [96, 97]. The onset and progression of both diabetes and obesity has been associated with oxidative stress which plays a prominent role in telomere shortening. Elevated levels of ROS produced as a result of oxidative stress make the G-rich regions of telomeric DNA susceptible to damage [98, 99]. This triggers DNA damage response pathways which causes telomere attrition [80]. Moreover, oxidative stress can activate telomerase reverse transcriptase (TERT), accelerating the degradation of telomeres. Another potential explanation is that oxidative stress induces cellular apoptosis or senescence, leading the surviving cells to undergo more frequent cell divisions, and expediting the process of telomere shortening. Hyperglycemia, buildup of AGEs, and insulin resistance have all been associated with telomere shortening [100]. Increased glucose uptake in endothelial cells of diabetic individuals is also reported to heighten oxidative stress leading to higher oxidative phosphorylation activity in the mitochondria. This oxidative stress can trigger a hyperglycemia-associated secretory phenotype (HASP) which initiates chronic-low-grade inflammation further elevating oxidative stress – a vicious cycle that leads to telomeric erosion [101]. Pro-inflammatory cytokines like IL-6 and TNF-α and C-reactive proteins in individuals with obesity also accelerate telomere shortening [102]. A high fat ratio in the muscles of obese people increases the circulation of free fatty acids. These fatty acids increase insulin secretion which eventually causes insulin-resistant and inflammatory phenotypes leading to shorter telomeres [103]. As a well-established mechanism, cancer cells favor shortened telomeres because of the moderate genomic instability elicited by them. This is because short telomeres induce end-to-end fusions of chromosomes and promote oncogenic transformations. Shorter telomeres were confirmed in breast cancer tissues than the normal epithelial cells [104]. Telomere shortening is also a classic model of progression from normal pancreas to adenocarcinoma where shorter telomeres in peripheral blood leukocytes are associated with an increasing risk of pancreatic cancer [105]. When evaluating the risk association of diabetes and obesity with cancer, the multifaceted role of shortened telomere length appears to emerge. The robust adaptive capacity of cancer cells to maintain a short telomere is advanced by diabesity and can be a plausible linkage between metabolic disorders and cancer.

## Role of gut microbiota in metabolic disorders

The human gut microbiota is a complex ecosystem including billions of microorganisms residing in the gastrointestinal tract, including viruses, bacteria, archaea, fungi, protozoa, and bacteriophages, which interact with the host in various ways [106, 107]. These microorganisms have a mass of approximately one and a half kilograms, thereby qualifying them as a 'microbial organ’ [108]**.** Recent findings indicate that the gut microbiota plays a vital role in maintaining metabolic health by engaging with dietary components to enhance nutrition processing, regulating energy utilization, and contributing to the biological synthesis of hormones, vitamins, amino acids, isoprenoids, and glycans [109]. Moreover, the gut microbiota influences the inflammatory response, regulates glucose and lipid metabolism, insulin sensitivity, and overall energy homeostasis in the mammalian host [107]. Alterations in gut microbiota composition have been associated with numerous metabolic disorders, including obesity, dyslipidemia, and T2DM [110].

The gut microbiome plays a significant role in energy metabolism, acting as a vital interface for energy intake by converting complex carbohydrates and fermenting dietary fibers that our body is unable to digest independently. They maintain the metabolic equilibrium within the host by modulating the concentrations of bioactive compounds like bile acids, short-chain fatty acids, ammonia, phenols, and endotoxins. These microbiota-derived metabolites facilitate microbe-host communication and are crucial for sustaining host physiology [111]. Short-chain fatty acids, such as butyric acid, acetic acid, and propionic acid, which are the final products of microbial fermentation of dietary fibers, modulate several metabolic processes. Propionate and butyrate facilitate glucose and energy homeostasis by stimulating intestinal gluconeogenesis and enhancing sympathetic nervous system activity. They may also improve the absorption of non-esterified fatty acids. Acetate and propionate may reduce intracellular lipolysis. Additionally, circulating acetate may be absorbed by the brain and influence satiety through the central self-regulation system. The cumulative impact of these factors may promote an elevation of triglycerides in adipose tissue while concurrently reducing the systemic release of free fatty acids [110]. Gut microbial dysbiosis disrupts these effects and increases occurrences of positive energy balance, such as obesity, where adipose tissue exceeds its buffering capacity and cannot store all excess energy as triglycerides. Butyrate also aids in decreasing gut permeability, suppressing appetite through the gut-brain axis, improving insulin sensitivity and energy metabolism, and is involved in fat oxidation by stimulating brown adipose tissue [112]. In support of this, Li et al. demonstrated that altering gut microbiota composition through every-other-day fasting (EODF) elevates acetate and lactate levels which stimulates beiging and may promote the activation of adipose tissue browning, thereby aiding in the treatment of metabolic disorders. Butyrate may boost human metabolism by increasing mitochondrial activity, reducing metabolic endotoxemia, and activating intestinal gluconeogenesis through the modulation of gene expression and/or hormone synthesis [108]. The gut microbiome also modulates the bile acid pool size in the host. The host generates a substantial, conjugated hydrophilic bile acid pool linked to pathological severities. This is often sustained through positive-feedback antagonism of the farnesoid X receptor (FXR) in the gut and liver. Microbiome members metabolize bile acids and their conjugates, leading to FXR activation in the colon and liver, which results in a reduced pool of unconjugated hydrophobic bile acids. Modification of the gut microbiome-bile acid-FXR axis is associated with obesity-related insulin resistance and hepatic steatosis in mice [109, 113, 114]. Evidence of microbiota-derived metabolites affecting glucose and lipid metabolism emphasizes their vital role in the advancement of metabolic diseases such as diabesity.

The gut 'superorganisms' have also been identified as a catalyst of metabolic inflammation, the pathophysiological foundation of diabesity. Certain gut bacteria, including R.intestinalis, B.fragilis, and L.plantarum, facilitate the synthesis of anti-inflammatory cytokines like IL-10, which may improve glucose metabolism, as the elevated presence of this cytokine in muscle tissue protects against age-related insulin resistance. R.intestinalis can augment IL-22 synthesis, an anti-inflammatory cytokine acknowledged for reinstating insulin sensitivity and alleviating diabetes. Beneficial microorganisms suppress pro-inflammatory cytokines and chemokines, thereby reducing inflammation. Multiple Lactobacillus species can reduce IL-1β, MCP-1, IL-8, and CRP levels [107]. Additionally, gut bacteria regulate the host's inflammatory response by maintaining the intestinal barrier function. The intestinal barrier safeguards the body from intestinal contents, and its impairment heightens permeability to bacteria or bacterial products, a process also known as metabolic endotoxemia, consequently resulting in chronic inflammation and metabolic disorders. Release of components of specific bacteria, such as cell capsule carbohydrates, lipopolysaccharides, and other endotoxins, leads to maintaining gut epithelium and thereby the integrity of the gut wall. Microbial dysbiosis in hyperglycemic and obese conditions may elevate the likelihood of intestinal barrier permeability, facilitating a ‘leaky gut’ and initiating an inflammatory response [106, 114, 115].

In healthy individuals, the gut microbiota is characterized by stability and diversity; a decline in these critical attributes may lead to disease and accelerate aging. Among the approximately 1000 bacterial species residing in the gut, nearly 90% are categorized within the phyla Bacteroidetes, comprising gram-negative bacteria, and Firmicutes, which consists of gram-positive bacteria. The Firmicutes/Bacteroidetes ratio represents a vital characteristic of dysbiosis that undergoes substantial alteration in individuals afflicted with diabesity, exhibiting significantly lower relative abundances of Firmicutes with a considerably larger proportion of Bacteroidetes [116, 117]. This ratio increases in association with factors such as BMI or fasting blood glucose levels. A human dietary intervention study suggested that weight loss in obese individuals correlates with an increased population of Bacteroidetes [109, 115]. Butyrate-producing microorganisms, notably from the Clostridiales order, are significantly diminished in patients diagnosed with T2DM [118]. Horne and colleagues discovered that a diet rich in fats and fructose modifies gut microbiome composition in Syrian hamsters, leading to dyslipidemia and hepatomegaly. Ruminiclostridium and Tyzzerella exhibited a positive correlation with fasting cholesterol levels, whereas Tyzzerella and the Ruminococcaceae NK4A214 group showed a positive correlation with fasting triglyceride levels [106]**.** The data presented further elucidate a robust correlation between gut microbiota and metabolic diseases.

## Potential for immuno-metabolic therapies in cancer

The progression of cancer therapy reflects a transition from conventional treatments to highly targeted, personalized, and advanced methodologies due to breakthroughs in molecular biology, immunology, and technological innovation. This encompasses the utilization of oncolytic viruses, epigenetic therapies, nanoparticle-based drug delivery systems, the application of artificial intelligence in the advancement of cancer nanomedicines, and immuno-metabolic therapies [119–121]. Immuno-metabolic therapies signify a noteworthy advancement in cancer treatment, focusing on the metabolic mechanisms present within the tumor microenvironment (TME). The TME is a complex entity comprising tumor cells, stromal cells, immune cells, blood vessels, and extracellular matrix. A dynamic interaction exists between cancer cells and TME components, wherein cancer cells engage with the surrounding cellular milieu and develop characteristics to enhance their survival [122, 123]. Cancer immuno-metabolic therapy leverages the functionality of immune cells to reconfigure the metabolic dominance of tumor cells and eradicate malignant cells. Immuno-metabolic therapies enhance classical immunotherapy by modifying immune cell metabolism to augment anti-tumor efficacy and mitigate immune suppression in the tumor microenvironment [124]. These therapies convert tumor-associated immune cells from a supportive function to an antagonistic function against cancers. One instance of this involves targeting metabolic processes, such as the TCA cycle, in anti-tumor immune cells, including cytotoxic CD8 + T cells and effector CD4 + T cells, to maintain their effectiveness. The pivotal enzyme of the TCA cycle, pyruvate dehydrogenase in CD8 + T lymphocytes, is targeted by pharmacological agents to preserve their functionality in the hypoxic conditions of the tumor microenvironment characterized by lactic acid buildup [125]. Moreover, the polarization of macrophages from the M2 (pro-tumor) to the M1 (anti-tumor) phenotype might substantially enhance immune activation [126]. The metabolism of tumor cells significantly depletes nutritional substrates, such as glucose and glutamine, in the tumor microenvironment, hence modifying the function of the immune cell population. The continuous cross-talk between immunological and metabolic signals in the tumor microenvironment, along with the influence of metabolic pathways on immune cells, highlights the importance of integrating immunometabolic signaling pathways. Regarding this, the LKB1-AMPK pathway serves as a crucial link between immunological signals and metabolic pathways, considerably influencing tumor growth. LKB1 plays a unique role in T-cell differentiation, and disruption of the LKB1-AMPK axis negatively impacts T-cell metabolism, resulting in the excessive stimulation of mTORC1 signaling, which promotes the proliferation of pro-inflammatory T cells [123]. Therapeutic targeting of specific immunometabolic pathways has the potential to transform the field of oncology.

## Combating diabetes and obesity—The cause-and-effect conundrum of cancer

The threat posed by diabetes, obesity, and cancer globally leaves us with the question of curbing them so that the incidence of the three can be regulated. Diet, physical activity, and behavior change are plausible lifestyle interventions that can improve the burden of these diseases. In this regard, intermittent fasting (IF) and short-term calorie-restricted diets have shown beneficial effects on lipid profiles and glycemic control in individuals with obesity and T2DM. IF has a crucial role in regulating proteins such as apolipoprotein A4, which promotes satiety, reverses cholesterol transport, and decreases oxidation of LDL-cholesterol particles. Time-restricted feeding, an advanced version of IF, is also thought to decelerate the development of tumors [127]. Adopting pharmacological approaches such as combinations of glucose-lowering drugs and those resulting in weight loss is the most accepted treatment if the disorders are acute or chronic. The question that now arises is whether these treatment processes influence the risk of cancer prognosis. Metformin, the chief drug involved in the treatment of diabetes has come into the limelight as an anti-cancer agent. The explanation for this can be its role in altering the energetics in cells and tissues of diabetic and cancer individuals through pathways involving AMPK. Metformin activates multiple anticancer pathways that are mediated by AMPK-dependent and AMPK-independent pathways [128]. Metformin also aids in tumor growth inhibition by protecting the CD8 + tumor-infiltrating lymphocytes (TIL), specific for tumor antigens, from immune exhaustion. Because of persistent tumor cells, CD8 + TILs lose their ability to produce triple cytokines TNFα, IL-2, and IFNϒ responsible for showing the PD-1-Tim-3 + phenotype, an effector memory subset causing tumor rejection. They are then eventually eliminated by apoptosis. Metformin increases the number of CD8 + TILs by restoring their multifunctionality thus inhibiting tumor growth [129]. Other anti-diabetic drugs, such as rosiglitazone, have been shown to have anticancer properties in breast cancer cell lines. Rosiglitazone has also been reported to reduce lung and colon carcinomas through PPAR-ϒ dependent pathways. Its anti-tumor activities include the prevention of tumor cell proliferation and cancer metastasis, diminishing immune resistance, promoting apoptosis, multidrug resistance reversion, induction of autophagy, and anti-angiogenesis [130]. Pioglitazone, insulin, GLP-1R agonists, sulfonylureas, glargine, and DPP4 inhibitors are among the anti-diabetic drugs linked to an increased risk of cancer. A correlation was found between elevated cancer onset and pioglitazone and insulin with its analogs, either intermediate or long-acting when combined with a fast-acting drug class in a study on the Taiwanese population. Pioglitazone was reported to be associated with a 53 percent increase in cancer risk [131].

The commencement of precision medicine has established a prominent role of anti-obesity medications in anti-cancer therapies. Statins are the most frequently used class of cholesterol-lowering medication that block HMG-CoA reductase, the rate-limiting enzyme of the mevalonate metabolic pathway. A statin, pitavastatin is reported to suppress chronic inflammation and subsequent development of cancers by blocking the TBK1-IRF3 pathway and effectively repressing the IL-33 inflammatory protein. The findings demonstrate that pitavastatin can be a potent preventive strategy to inhibit cancer-prone chronic inflammation [132]. Prior research has also shown that statins effectively disrupt estrogen receptor expression, cancer cell survival, and proliferation by blocking the mevalonate pathway, thereby reducing breast cancer progression [133]. Statins along with doxorubicin expressed a synergic effect in reducing cancer progression in a xenograft model of metastatic triple-negative breast cancer [134]. Meta-analysis of several other anti-obesity drugs such as phentermine/topiramate, liraglutide, lorcaserin, naltrexone/bupropion, and orlistat have shown effective weight loss reduction. However, lorcaserin is debated to be associated with an increased risk of cancer. In a random, placebo-controlled, double-blind trial, a greater number of lorcaserin-treated participants were diagnosed with higher rates of colorectal, pancreatic, and lung cancer as compared to the placebo group [135]. Therefore, drug therapy for both diabetes and obesity should be started after a careful evaluation of their carcinogenic effects and associated benefits and risks.

## Conclusion

As understood from the current review, several intracellular biological pathways are shared in metabolic antipathies like diabetes, and obesity which promote oncological complications. We show here that metabolic reprogramming of multiple pathways involved in hyperglycemic and inflammatory events of diabesity ultimately leads to the onset of various cancers. Oxidative stress and altered hormonal landscapes in diabesity contribute to DNA damage and telomere length attrition paving the way for genomic instability and augmenting tumorigenesis. We tried to integrate some of the substantial biochemical and molecular factors that link diabetes, obesity, and cancer. Discussions on the paradoxical role posed by the biochemical pathways reflect the importance of understanding the specific roles of various intermediates involved in signaling cascades shared among the three diseases. The exploration of gut microbiota and immunometabolic therapies introduces an intriguing dimension to the interrelation of the three disorders. Exploratory studies on how the expression of diabesity-related signaling protein varies under wavering glycemic levels and adiposity indices and decisive data on the precise mechanisms of their interactions in the development and/or maintenance of malignancies need to be addressed. There may be arrays of biological mechanisms beyond the scope of this review promoting tumorigenesis in patients with diabetes and obesity. Each of these needs to be understood through in silico, in vitro, and in vivo studies to develop a holistic treatment approach.

## Data Availability

No datasets were generated or analysed during the current study.

## Abbreviations

4-HNE: 4-Hydroxy-2-nonenal
8-OHdG: 8-Hydroxy-2' -deoxyguanosine
8-oxodG: 8-Dihydro-2-deoxyguanosine
PKB: Protein kinase B
AMPK: Adenosine monophosphate-activated protein kinase
APC: Adenomatous Polyposis Coli
ATM: Ataxia telangiectasia mutated
ATR: Ataxia telangiectasia and Rad3-related protein
BER: Base Excision Repair
BMI: Body Mass Index
BRCA: BReast CAncer gene
cAMP: Cyclic adenosine monophosphate
CD4: Cluster of differentiation 4
CD8: Cluster of differentiation 8
CK1: Casein kinase 1
CRP: C-reactive protein
CSA: Cockayne Syndrome type A
CSB: Cockayne Syndrome type B
DAAM1: Disheveled associated activator of morphogenesis 1
DMBA: 712-Dimethylbenz(α)anthracene
DNA: Deoxyribonucleic acids
DPP4: Dipeptidyl peptidase-4
ERK: Extracellular signal-regulated kinase
GLP-1R: Glucagon-like peptide-1 receptor
GLUT4: Glucose transporter protein type-4
GOAT: Ghrelin O-acyltransferase
Grb2: Growth Factor Receptor-bound protein 2
GRK: G protein-coupled receptor kinase
GSH: γ-Glutamylcysteinyl glycine
GSSG: Glutathione disulfide
GSK3: Glycogen synthase kinase 3 protein
HDL: High-density lipoprotein
HIF-1α: Hypoxia-inducible factor
HMG-CoA: Hydroxymethylglutaryl-CoA
IGF: Insulin growth factor
IL-6: Interleukin
IR: Insulin receptor
IRF3: Interferon regulatory factor 3
IRS: Insulin receptor substrate
JAK: Janus kinase
JNK: JUN N-terminal kinase
LKB1-AMPK: Liver kinase B1–5′ AMP-activated protein kinase
LRP 5/6: Low-density lipoprotein receptor-related protein
MAPK: Mitogen-activated protein kinase
MCP-1: Monocyte chemoattractant protein-1
MCT-1: Monocarboxylate transporter 1
MDA: Malondialdehyde
MMP-7: Matrix metalloprotein-7
MRLC: Myosin Regulatory Light Chain
mTOR: Mammalian target of rapamycin
mTORC1: Mammalian target of rapamycin complex 1
NADPH: Nicotinamide adenine dinucleotide phosphate hydrogen
NER: Nucleotide excision repair
NF-κβ: Nuclear factor kappa-light-chain-enhancer of activated B cells
NHEJ: Non-homologous end joining
OGG1: 8-Oxoguanine glycosylase
PCP: Planar cell polarity
PDK: Pyruvate dehydrogenase kinase
PHD: Prolyl hydroxylase domain proteins
PI3K: Phosphoinositide 3-kinase
PKA: Protein kinase A
PKC: Protein kinase C
RAF: Rapidly accelerated fibrosarcoma kinases
RAP2A: GTPase Ras-related protein 2
RAS: Rat sarcoma
ROCK: Rho-associated protein kinase
ROR2: Receptor Tyrosine Kinase-Like Orphan Receptor 2
ROS: Reactive oxygen species
STAT: Signal transducer and activator of transcription
TBK1: TANK-binding kinase 1
TCF/LEF: T cell factor/lymphoid enhancer factor
TLR-4: Toll-like receptor 4
TNF-α: Tumour necrosis field-α
TRIB1: Tribbles homolog 1
UCP2: Uncoupling protein 2
VEGF: Vascular endothelial growth factor
Wnt: Wingless-related integration site
XPA: Xeroderma pigmentosum complementation group A
XPC: Xeroderma pigmentosum complementation group C
